# Laryngeal Foreign Body Causing Bronchial Spasm

**DOI:** 10.1016/S1808-8694(15)30090-2

**Published:** 2015-10-19

**Authors:** Giulliano Enrico Ruschi e Luchi, Jose Jarjura Jorge Junior, Cassio Caldini Crespo, Jaime Nakasima, Fabio Eduardo Caramante Pizzini, Rogerio Poli Swensson, Pedro Robson Boldorini

**Affiliations:** a Otolaryngologist, MSc student at the School of Medical Sciences - Santa Casa de São Paulo; b PhD in Otolaryngology, Full Professor of Otolaryngology at PUC-SP; c Otolaryngologist, Professor of Otolaryngology at PUC-SP; d Otolaryngologist; e Otolaryngologist; f Resident; g Otolaryngologist. Pontifícia Universidade Católica de São Paulo PUC-SP

**Keywords:** laryngeal foreign body, recurrent bronchial spasm

## INTRODUCTION

Foreign bodies (FB) in the larynx and trachea usually lead to alarming circumstances, with death by complete obstruction of the respiratory tract as its utter consequence. Incidence rates are not high (2-11% of respiratory tract foreign bodies). Children from 6 months to 4 years of age are the most frequently affected, as expected due to their yet immature swallowing neuromuscular mechanism and the oral stage. In general terms, inhaling FB produces intense coughing and suffocation, and possibly cyanosis, asphyxia, sweating, vomiting, and dysphonia. Larynx papillomatosis must be ruled out as it may present similar findings 1, 2, 3.

## CASE STUDY

R.A, 10 years old, male, choked two months earlier while eating fish. His mother reported spontaneous improvement, however with onset of hoarseness. A few days later he began to wheeze and developed dysphonia. Systemic corticosteroids, mucolytic agents, and inhaler with bronchodilators were administered and led to partial reduction of wheezing.

Two months later recurrent dyspnea and elimination of blood clots led the mother to take him to see an otolaryngologist. Back then the patient was eupneic, acyanotic, and made no special effort while breathing. Flexible naso-laryngofibroscopy identified a FB in the anterior commissure. The patient underwent direct laryngoscopy under general anesthesia on the same day to remove the FB. On the following day a new exam showed reduced mobility in the patient’s right vocal cord and a small granuloma in the anterior commissure. He was kept on systemic corticosteroids and antibiotics. On day 13 significant improvement was found in the patient’s vocal patterns and absence of oropharyngeal complaints. Vocal cords were back to normal mobility and the granuloma in the anterior commissure persisted.

**Figure:**
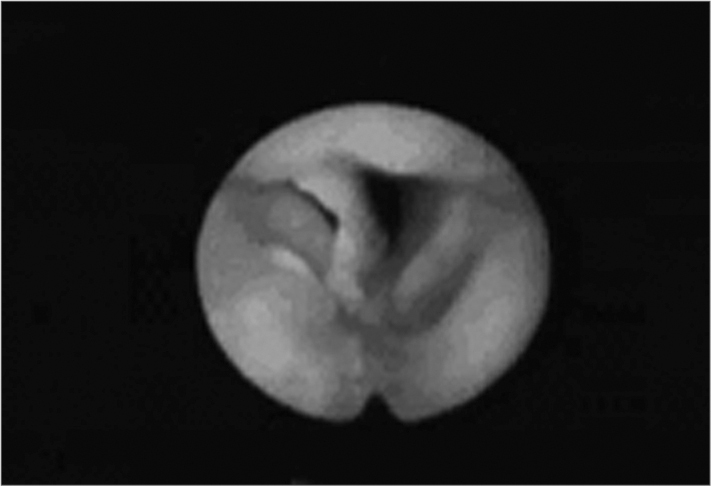
**Foreign body in the larynx -** laryngoscopy image showing foreign body in the glottic region.

## DISCUSSION

As mentioned before, FB in the larynx is more frequent in children. The foreign bodies themselves can be either organic or inorganic. In the case reported the patient choked on a fishbone fragment. Usually dysphonia, dyspnea, wheezing, snoring, strenuous and labored breathing occur in previously healthy children. The clinical history of the patient began with choking followed by persistent dysphonia and wheezing managed with bronchodilators that allowed for two months of partially improved symptoms 3, 4, 5.

Yadair published a case of larynx FB in which the diagnosis was reached after four months into follow-up. Once the possibility of larynx FB is raised, the larynx must be thoroughly examined by direct or indirect laryngoscopy. Naso-fibrolaryngoscopy was performed right off the start, thus providing diagnostic confirmation 5, 6.

Most publications in the literature refer to FB removal under general anesthesia as the management of preference, using suspension laryngoscopy or bronchoscopy, as the foreign body may move onto inferior airways leading to asphyxia. The chosen approach was removal under general anesthesia through suspension laryngoscopy ^5^.

## CONCLUSIONS

In cases such as this, one must be careful when using pre-defined diagnosis and should always look for information in the interview that may lead to proper case identification. As the foreign body’s presence in the larynx is confirmed, an expert assisted by a colleague skilled in handling the bronchoscope must remove it with the patient under general anesthesia.
